# Transfer after Working Memory Updating Training

**DOI:** 10.1371/journal.pone.0138734

**Published:** 2015-09-25

**Authors:** Otto Waris, Anna Soveri, Matti Laine

**Affiliations:** 1 Department of Psychology, Åbo Akademi University, Turku, Finland; 2 Turku Brain and Mind Center, Turku, Finland; Centre de Neuroscience Cognitive, FRANCE

## Abstract

During the past decade, working memory training has attracted much interest. However, the training outcomes have varied between studies and methodological problems have hampered the interpretation of results. The current study examined transfer after working memory updating training by employing an extensive battery of pre-post cognitive measures with a focus on near transfer. Thirty-one healthy Finnish young adults were randomized into either a working memory training group or an active control group. The working memory training group practiced with three working memory tasks, while the control group trained with three commercial computer games with a low working memory load. The participants trained thrice a week for five weeks, with one training session lasting about 45 minutes. Compared to the control group, the working memory training group showed strongest transfer to an n-back task, followed by working memory updating, which in turn was followed by active working memory capacity. Our results support the view that working memory training produces near transfer effects, and that the degree of transfer depends on the cognitive overlap between the training and transfer measures.

## Introduction

Working memory (WM) refers to a temporary memory storage system that maintains and manipulates currently active information [1]. WM is related to many cognitive capabilities such as reasoning skills [2], mental focus in everyday activities [3], and multitasking [4]. WM has also been linked to academic achievement as well as developmental and neuropsychiatric conditions such as learning disorders and ADHD (for a review see e.g., [5]). Improved WM through training could therefore, in principle, have wide-reaching positive effects on cognitive function, and WM training has been a popular and debated research topic during the past decade (for reviews, see e.g., [6–9]; for discussions regarding the commercial CogMed training program see e.g., [10–13]).

Cognitive training studies have consistently shown improvement on the trained task(s), but the main goal of cognitive training is its generalization (transfer) to untrained measures of cognitive function and even to everyday behavior. Transfer is commonly divided into “near” and “far” transfer. Near transfer indicates improvement on an untrained task within the trained cognitive domain (e.g., WM), while far transfer indicates improvement on a task within another cognitive domain (e.g., fluid intelligence) that shares some cognitive component(s) with the trained domain. It has been suggested that transfer can occur when the training and transfer measures activate overlapping brain regions [14–15].

WM training outcomes have been variable regarding transfer effects: some studies have indicated that near, or both near and far transfer effects are achievable, while others have found no signs of transfer (e.g., [16–27]). A recent meta-analysis indicated that WM training produces specific short-term gains that do not generalize beyond WM [28]. However, another meta-analysis studying the effects of n-back WM training on fluid intelligence indicated a small net effect gain in *Gf* outcome measures [29]. Considering the present lack of consensus regarding the cognitive effects of WM training, further studies are warranted.

In the present WM training study, we focused especially on training the flexible updating of WM contents according to task demands (see e.g., [30–31]). On the basis of the multicomponent model of WM by Baddeley and Hitch [1, 32] and the factorial model of executive functions (WM updating, inhibition, and set shifting) by Miyake et al. [31], one can delineate aspects of WM function that could be affected by WM training. These include passive (only involving maintenance) and active (maintenance and manipulation) WM capacity, (see e.g., [33–35]), the updating function, shifting between mental sets, and the inhibition of irrelevant material during WM processing. All of these aspects of WM performance were assessed in the present study. Additionally, fluid intelligence and dual-tasking were assessed, as these functions have been related to WM performance (for WM and fluid intelligence see e.g., [2, 36]; for WM and dual-tasking see [37]).

The current training regime included three training tasks. A diverse training regime possibly strengthens the transfer effects, as a multiple-paradigm approach should go beyond task-specific training effects and target the WM updating function on a latent level [38]. Additionally, a diverse training regime possibly increases motivation and engagement for all participants. However, the drawback is the difficulty in determining how individual training tasks affect the outcomes.

As some of the previous WM training studies have been criticized for methodological shortcomings regarding the use of a passive or nonadaptive control group and utilization of single measures to evaluate cognitive constructs (e.g., [7, 39]), we employed a control group with a primarily adaptive training regime and measured the cognitive constructs with composite scores. Additionally, given the great variability in transfer measures and outcomes in earlier studies, we explored whether our extensive pre-post test battery would reveal a systematic pattern of transfer effects. In other words, we expected that the magnitude of transfer would be related to the cognitive overlap between the training tasks and a given pre-post measure. We did not hypothesize a specific ranking order for the transfer effects, but expected that the pre-post measures being most similar to the training regime in terms of their cognitive demands would show strongest transfer. One would, for example, expect a trained criterion task to show the strongest signs of training and near transfer measures to show stronger transfer effects than far transfer measures. In summary, our main aims were as follows: 1) to search for transfer effects of WM updating training with a setup that takes into account recent methodological criticisms and 2) to evaluate the transfer gain effect sizes of the pre-post measures on the basis of the notion that greater cognitive overlap between the training and transfer measures should produce greater transfer.

## Materials and Methods

### Ethics Statement

This study was approved by the Institutional Review Board of the Department of Psychology and Logopedics, Åbo Akademi University, and written informed consent was obtained from all participants.

### Participants

The sample consisted of 31 (27 women) healthy Finnish adults in the age range of 19–30 years (*M* = 24.16, *SD* = 3.28). All the participants had passed the Finnish matriculation exam or were currently studying at an undergraduate level. The participants did not report any significant psychiatric or neurological illnesses. All participants completed the WAIS-III Similarities subtest [40], the Beck Depression Inventory II (BDI-II [41]), and a background information questionnaire during pretesting. Participants with BDI-II scores exceeding the cut-off score of 16 were excluded from the study. The participants were randomized into either a WM training group or an active control group, but were not informed of the existence of the two groups. The groups were comparable regarding age, *t*(29) = 0.921, *p* = .365, gender χ^2^(1, *N* = 31) = 1.006, *p* =. 316, WAIS-III Similarities scaled scores, *t*(29) = 1.558, *p* = .130, and BDI-II scores, *t*(29) = 0.852, *p* = .401 (see Table 1). All participants received a compensation of 70 euros.

**Table 1:** Descriptive data on the study groups.

	WM training group	Active control group
Age	23.6 (3.8)	24.7 (2.7)
Sex F/M	14/1	13/3
WAIS-III Similarities Scaled Scores	12.5 (1.4)	11.8 (1.4)
BDI-II	4.2 (3.1)	3.3 (2.7)
Motivation: Beginning of training	8.6 (1.1)	7.8 (1.5)
Motivation: Halfway through training	7.3 (1.4)	7.8 (1.9)
Motivation: In general	7.2 (1.5)	7.4 (1.3)
Improvement on training tasks	7.5 (2.0)	6.9 (1.1)

Age in years, gender distribution, scaled scores on the WAIS-III Similarities subtest, BDI-II scores, and retrospective evaluations of motivation and level of improvement on the training tasks (SDs in parentheses).

### Procedure

All participants took part in pre- and posttest sessions. The posttest session included the same tests (see below) as the pretest, except for the WAIS-III Similarities and the background questionnaire. Moreover, at the end of the posttest session, the participants retrospectively evaluated their motivation at the start of training, halfway through the training, and for the whole training in general. They were also asked to evaluate how much they had improved on the training tasks. All questions were rated from one (not at all motivated/no improvement) to ten (very motivated/improved very much). One to three participants were tested simultaneously in a single room with screens blocking visual contact between the participants. The test order was randomized for each separate testing session. Pretesting lasted for two-and-a-half to three hours, while the posttest took about two hours.

Training commenced during the week following the pretest session. The participants trained three times (45 min each) a week for five weeks, after which they completed the posttest session during the following week. The WM training group practiced with three computerized WM tasks (see below), while the active control group trained with three commercial computer games that taxed WM only slightly. The order of the training tasks was randomized for every training session. The participants recorded their individual training performances for each training session in personal training logs.

### The pre- and posttests

The various aspects of WM function listed above were tapped with the following measures that were selected on the basis of task features and previous research. Additionally, the test battery included a Selective Updating task (SUT, a criterion task trained by the WM training group), and a visual numerical n-back task. **WM updating**: a Verbal Running Memory task (VRM) and a Visuospatial running memory task (part 1 of the VisMem task, see below). **Active WM capacity**: Digit span backward and Corsi Block Task (CBT) backward. **Passive WM capacity**: Digit span forward and CBT forward. **Set shifting**: a Number-Letter task and a visual sorting task (included in parts 2–3 of the VisMem). **Dual task performance**: a Visuospatial Memory task with simultaneous sorting (parts 2–3 of the VisMem). **Fluid intelligence**: subtests two and four from the Cattell Culture Fair Intelligence Tests (CFIT [42]). **Inhibitory control**: This composite was not included as our Simon task [43] turned out to show ceiling effects (the Simon effects of both the pretest RT in milliseconds [*M* = 15.05, *SD* = 27.84] and the accuracy rate in percentage correct [*M* = 0.74, *SD* = 1.67] were negligible to begin with) and technical issues resulted in considerable data loss in the inhibitory control measure derived from a Number Substitution task [44] (the data of five participants in the active control group and one participant in the WM training group were lost). The omitted tasks will not be described in more detail.

Two sets of test items were created for the VRM, the VisMem, the SUT, and the n-back task, and the participants were counterbalanced across sets: half of the participants received one set of items during the pretest and the other set during the posttest, while the order was reversed for the other half of the participants. In the CFIT, forms A and B were used as separate versions, and the participants were counterbalanced across these versions.

#### Selective Updating Task

**Fig 1:**
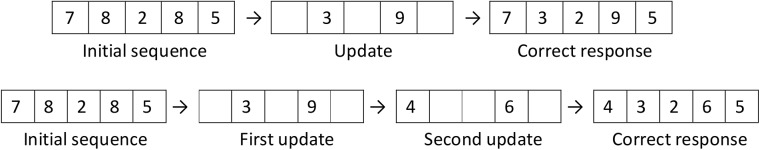
Example sequences of the SUT with one (upper row) and two (lower row) update stages.

The SUT is a WM updating task based on Murty et al.’s [45] task design. In our version, five numbers from zero to nine were presented on the computer screen in a row of five boxes. The participant was to memorize the number sequence, that is, the correct number for each box. After this, the initial number sequence disappeared and a new row of five boxes was displayed. Two of the new boxes contained numbers, while three were empty. The participant had to replace the old numbers with the newly presented numbers, while also maintaining the remaining unchanged numbers in WM, thus creating a new number sequence. Depending on the task sequence, there were zero (baseline), two, three, or five update stages (i.e., replacement of old numbers with new numbers). After all the update stages, the participant was to report the final number sequence (see Fig 1).

One point was awarded for every correctly placed number. Results were calculated separately for accuracy at baseline vs. update (the 2, 3, and 5 update stage trials were pooled). An updating effect was then calculated by subtracting the accuracy (in percent) of the update items from the accuracy of the baseline items, and this measure was used as the outcome variable.

When creating the test versions, the initial number sequences were randomly generated as were the update positions and numbers, except that the old and new numbers were not allowed to be identical (e.g., a 3 replacing a 3 in the same box). Each version consisted of ten baseline trials, ten trials with two updates, ten trials with three updates, and ten trials with five updates. The order of the trials was randomized for each participant, and the participants were unaware of how many, if any, update stages the current trial incorporated. The initial number sequence was shown for 4000ms, followed by a 100ms blank screen, after which the update stage was presented for 2000ms. The update stage was once again followed by a 100ms blank screen and the next update stage. After all the update stages had been presented (none in the baseline condition), an empty row of five boxes appeared. The participant responded by pressing the number buttons on the computer keyboard.

#### N-back task

Introduced by Kirchner [46], the n-back task has been widely used as a WM updating measure. The n-back task in this study included so-called lure trials in order to encourage active WM updating instead of familiarity matching [47]. In the current n-back task, numbers from one to nine were presented one at a time on a computer screen. Depending on the task, the participant had to press a “yes” button if the currently presented number was the same as the number preceding it (1-back task) or the one presented three numbers back (3-back task). For the remaining items the participant had to press a “no” button.

Both pre- and posttests consisted of two blocks of 1-back trials and eight blocks of 3-back trials that were administered in a random order. Each block included 48 items that required a response. Half of the blocks included lure trials. A lure trial in the 1-back task was an n +1 item, that is, an item matching the item presented two numbers back (e.g., in the 1-back sequence 2-3-4-3, the last number “3” is a n+1 lure). The 3-back task lure trials consisted of both n+1 (current item matches the item presented 4 numbers back) and n-1 (current item matches the item presented 2 numbers back) lures. An n-back effect, which was used as the outcome variable, was calculated by subtracting 3-back accuracy (in percent) from 1-back accuracy.

Each block began with instructions of the task type (1- or 3-back) and which response keys to use. These instructions were visible for 4000ms, after which a fixation point appeared in the middle of the screen. After 450ms, the fixation point was replaced by a number that was visible for 1500ms. The alternation of a fixation point and a number continued until the end of the block, after which the instructions for the next block appeared. The participants had 1950ms (number + fixation point) to respond to each item. The number sequences in every block were pseudorandomized in order to ensure the exclusion of trial types that were not allowed in the specific condition (i.e., n+1 trials in the 1-back no lure condition, n-1 and n+1 trials in the 3-back no lure condition, and n-2 trials in either 3-back condition).

#### Verbal Running Memory task

The VRM was developed to measure verbal WM updating. Sequences of words were presented on the screen, and at the end of each sequence the participant was to report the last four words (targets) in the presented order.

The task included 26 trials of which eight trials contained semantic foils, eight trials phonological foils, eight trials no foils, and two trials were catch trials. The participants were not informed of this feature. The semantic foil trials contained two words within the pre-target words that were semantically related (e.g., foil donkey–target pony) to two of the target words, while the phonological foil trials contained two words that deviated only by a single letter from two target words (e.g., the Finnish words taso “level”–talo “house”). These foils always appeared directly before the four target words in every sequence. The foil conditions were expected to increase demands on the management of WM interference in the whole task. The catch trials were four-word sequences that were placed at the beginning and at the end of the test. They were intended to enforce active memorization from the start of each sequence (the catch trials were not included in the analyses). One point was awarded for every correctly reported word, and the total score was used for the WM updating composite score.

The sequence length varied from seven to fourteen, except for the two four-word catch trials. Each trial type (semantic foils, phonological foils and no manipulations) contained one trial of each sequence length (7–14), giving a total of three trials per sequence length. The trial order was randomized (except for the catch trials), and the participants were unaware of the sequence length of a trial. The test began with two seven-word no-lure practice trials, after which the actual test commenced. Every trial began with the text “the sequence commences”. After 2000ms, the text was replaced by a fixation point that appeared in the middle of the screen. The fixation point was visible for 1000ms, and it was followed by a word that was visible for 1500ms. The alternation of a fixation point and a new word continued until the end of the sequence, whereupon the response screen was displayed. It consisted of four horizontally aligned empty boxes, and the participant was to type his/her response in each box. The next trial started automatically when the fourth and final word had been entered.

Bi-syllabic four-letter nouns of varying frequencies were used. Words were selected from a massive Finnish newspaper corpus with 22.7 million word tokens by using the WordMill lexical search program [48]. A total of 398 words were selected of which 260 words were separately pseudorandomized for the two test versions. Each word appeared only once during each test version. Identical words were allowed to appear in the two test versions with the exception that the same foil-target word pairs were never used in both test versions. One-way ANOVAs were used to compare the lemma, surface, and bigram frequencies of the target words and the two words preceding the target words. These analyses revealed no significant differences between the stimulus types in either test version (*F* range 0.05–1.15, *p* range .949 - .333).

#### The VisMem task

**Fig 2:**
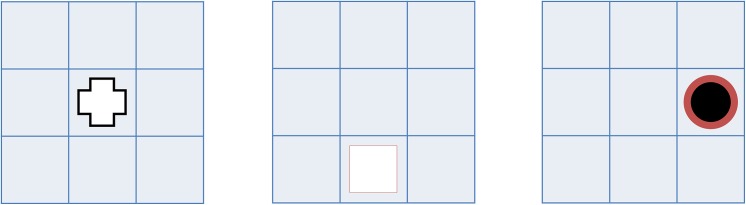
Examples of the VisMem stimuli. From left to right: VisMem–updating cross, VisMem–single task or switch trial white square with thin borders (sort by shape), and VisMem–single task or switch trial black circle with thick borders (sort by color).

The VisMem task was specifically developed for this study. Part one demands WM updating, while parts two and three call for simultaneous visuospatial WM updating and sorting, resulting in a dual task performance. The objective of the task was to constantly maintain in WM the four last locations of a figure in a 3x3 matrix (part 1), while simultaneously (parts 2–3) sorting the figures according to simple sorting rules (see Fig 2). The VisMem consisted of three parts that were always performed in the same order. All parts consisted of two practice trials and eight actual sequences. The practice trial sequence length was seven for both trials in all parts. The actual sequences were seven to fourteen items long, and the order of the sequences was randomized. One sequence of each length was included. The participants were unaware of the sequence length.

1. VisMem–updating. The first part only contained the visuospatial running memory element. A white cross appeared inside a 3x3 matrix, and the participant was to keep in mind the four last locations where the cross appeared. Every sequence began with an empty matrix. After 500ms, the cross appeared inside one of the nine locations. After 1500ms, the cross disappeared and the matrix was blank for 500ms. An alternation of cross and blank matrix continued until the end of the sequence, whereupon the participants were to recall, in correct order, the four last locations of the cross. Locations were chosen by using the computer mouse. The selected locations flashed blue for 100ms.
2. VisMem–single sorting rule. The second part encompassed both the visuospatial running memory and sorting elements. Figures appeared inside the 3x3 matrix. The participant was to keep in mind the four last figure locations and to sort the figures according to shape (circle or square) or color (black or white) every time a figure appeared. The thickness of the figures’ red border indicated which sorting rule to implement: thick borders indicated shape and thin borders indicated color. The same sorting rule was always implemented during a single sequence and the participant was informed of this rule. The presentation times were the same as for part 1, except that each figure was shown for 2000ms instead of 1500ms. The participant was to sort each figure immediately when it appeared. The sorting responses were given by pressing the mouse buttons: left button for black color or square and right button for white color or circle.
3. VisMem–mixed sorting rules. This part was identical to the second part, except for switches in the sorting rule during the sequences (the participant was informed of this rule). The sequences with seven to ten figures contained two switches, while the sequences with eleven to fourteen figures contained three switches. This condition was expected to provide a pre-post measure of the interplay of set shifting and WM updating.

For each part of the test, the total number of correct item-by-item spatial locations was calculated. The score from part one was used for the WM updating composite score, while the scores from parts two and three were used for the dual task performance score. RTs and response accuracies were also calculated for the sorting/set shifting element in parts two and three, and the mixing and switching costs (see the Number-Letter task for details) were calculated from these results and used for the set shifting composite score.

#### Computerized Corsi Block Task

A computerized CBT [49] was developed for this study. The task requires visual attention and visuospatial WM. In the forward version, the participant must press the squares in the exact order in which the squares are lit (turn yellow) on the computer screen. In the backward version, the participant must press the squares in the opposite order.

A practice trial (span = 2) was administered at the beginning of each task version. The practice trial was repeated until the participant answered correctly. Both forward and backward tasks began with a span of two, with a maximum span of nine in the forward version and eight in the backward version. Two trials of each span length were administered. The span length was increased by one if the participant answered correctly on either trial of a specific span length. The test was discontinued if the participant answered incorrectly on both trials within a specific span length. One point was awarded for every correctly recalled sequence, and the total scores for each test version were included in their respective capacity composite scores.

Ten blue squares were visible on the screen. After 500ms, one of the squares turned yellow. The square was yellow for 1000ms, after which it once again turned blue. After 500ms, another square turned yellow. The lighting of squares continued until the end of the trial. A given square turned yellow only once (if at all) during a single trial. At the end of the sequence, a small green circle appeared in the middle of the computer screen that prompted the participant to respond. The green circle was visible until the participant finished responding. The participant selected the squares by using the computer mouse and pressing the left mouse button. Selected squares flashed yellow for 100ms. The participant pressed the Enter button when the selection was completed.

#### Computerized Digit span task

A visuoverbal equivalent to the CBT, the Digit span task provided a pre-post measure of verbal WM capacity. Number sequences were presented to the participant, who was to report back the numbers in the same (forward version) or opposite (backward version) order.

Both forward and backward tasks began with a span of two, with a maximum span of nine in the forward version and eight in the backward version. Two trials of each span length were administered. The span length was increased by one if the participant answered correctly on either trial of a specific span length. The test was discontinued if the participant answered incorrectly on both trials within a specific span length. One point was awarded for every correctly recalled sequence, and the total scores for each test version were included in their respective capacity composites.

Each sequence began with a fixation cross in the middle of the screen that disappeared after 1000ms. The screen was blank for 1000ms after which the first digit (1–9) was presented. The digit was visible for 1000ms, and it was followed by another 1000ms blank screen interval. The alternation of a number and a blank screen continued until the end of the sequence, whereupon the text “Type your answer” appeared. There was no time limit for typing in a response.

#### The Number-Letter Task ([50], adapted from [51])

This task was implemented as a far transfer measure for set shifting. Number-letter pairs were presented in the center of the screen in one of two vertically aligned boxes. If the number-letter pair appeared in the upper box, the participant was to determine if the presented number was odd or even. If the number-letter pair appeared in the lower box, the participant was to determine if the letter was a vowel or a consonant. Only two response buttons were used: one for even number or vowel and the other for odd number or consonant.

Every trial began with a blank screen. After 150ms, a fixation cross appeared in the middle of the screen. The fixation cross was replaced by two vertically aligned boxes after 300ms. One of the boxes contained a number-letter pair that was visible until a response was given or until 3000ms passed, after which the next trial commenced. The task included two single-task blocks (32 trials each) and a mixed-task block (80 trials). During the single-task blocks the sorting rule (odd-even or vowel-consonant) never changed. In the mixed-task block the trials were divided into no-switch (48 trials) and switch trials (32 trials). On the no-switch trials the number-letter pair appeared in the same location as the previous trial; therefore the same sorting rule was to be implemented. On the switch trials the location of the number-letter pair changed; consequently a switch of the active sorting rule was required. All three blocks were preceded by a 16-trial practice sequence that was repeated once if the participant made any mistakes.

Mixing and switching costs were calculated as dependent variables separately for the percentage of correct responses and mean RTs. The mixing cost accuracy effect was calculated by subtracting the mean of the no-switch trials from the mean of the single-task trials, while the mixing cost RT effect was calculated by subtracting the mean of the single-task trials from the mean of the no-switch trials. The mixing cost is considered to measure the cognitive load of keeping two sorting rules active compared to only one [52]. The switching cost accuracy effect was calculated by subtracting the mean of the switch trials from the mean of the no-switch trials, while the switching cost RT effect was calculated by subtracting the mean of the no-switch trials from the mean of the switch trials. The switching cost gives a pre-post measure of the cognitive load of switching mental sets/rules [53].

#### Culture Fair Intelligence Tests

The CFIT were employed as a far transfer measure of fluid intelligence and visual reasoning. In this study, subtests two (Classification) and four (Conditions) from Scale 3 were used. In subtest two, five figures are presented, and the participant must choose two figures that differ somehow from the other three. In subtest four, the participant has to choose among five alternatives the one that duplicates a set of conditions. The total score was used as the outcome variable.

### The training tasks for the WM training group

#### Selective Updating training task (SUTT)

This task was very similar to the pretest SUT. A minor alteration was the duration of the update stages that was 4000ms in the SUTT compared to 2000ms in the SUT. The major difference was the progressive level of difficulty that was implemented in the training task. Eleven difficulty levels were created (baseline + 10 levels). The baseline level consisted of five initial numbers and no updates, with one added update for every increment in the difficulty level up to level six (5 numbers and 6 updates). Level seven included six numbers and two updates, and once again one update was added with every increment in the level of difficulty. The maximum difficulty level was ten, which included six initial numbers and five updates.

Every training session with the SUTT included a four-minute practice phase with the previous difficulty level and a ten-minute training phase with the current difficulty level (starting with a four-minute baseline phase and a ten-minute level one phase). The difficulty level was raised by one (for both practice and training phases) for the next training session if at least 80% of the sequences (i.e., 5 or 6 correct numbers) during the ten-minute training phase were correct. If the participant reached difficulty level seven, the four-minute practice phase always consisted of level six, with ten minutes of level seven to ten depending on how far the participant advanced. The difficulty level either increased or remained unaltered. Percentage of correct items and sequences were reported to the participants separately for both the four- and ten-minute phases at the end of the SUTT session, and the participants recorded these results in their personal training logs.

#### Moving Figures

**Fig 3:**
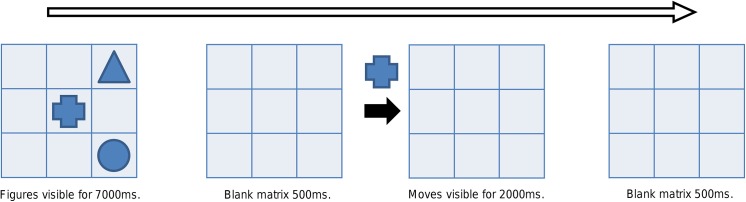
Example of a Moving Figures task with three figures. Only one move is shown in this example while an actual sequence consisted of several turns, each consisting of one to three moves.

This training task was created specifically for this study to tap visual and visuospatial WM with an emphasis on WM updating. The participant was to memorize the location of multiple figures inside a matrix and to keep track of their locations while they moved. The challenge of the task was that the figures were not visible inside the matrix while they moved; so their locations had to be maintained mentally. The figure that moved was shown on the left side of the matrix, while the direction of movement (one step within the matrix) was signaled by an arrow that was located underneath the figure (up, down, left, or right, see Fig 3). Each figure moved once, twice, or thrice in a row during each of its “turns”. The figure turns and the direction of each move were randomly generated; however, the same figure could not have consecutive turns and the figures were not allowed to move outside the matrix. The figures could, however, move on top of each other. At the end of the sequence, the participant was to indicate where each figure was currently located.

**Fig 4:**
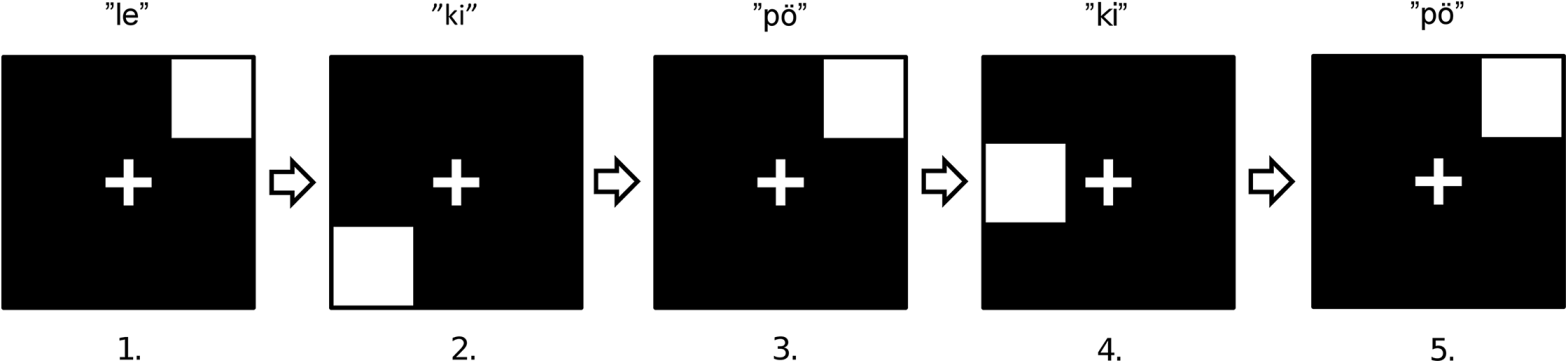
Example of a short dual 2-back sequence. The aurally presented syllables are written above the matrix in this example. The left response button (location match) should be pressed on the third and fifth trials, while the right response button (syllable match) should be pressed on the fourth and fifth trials.

**Fig 5:**
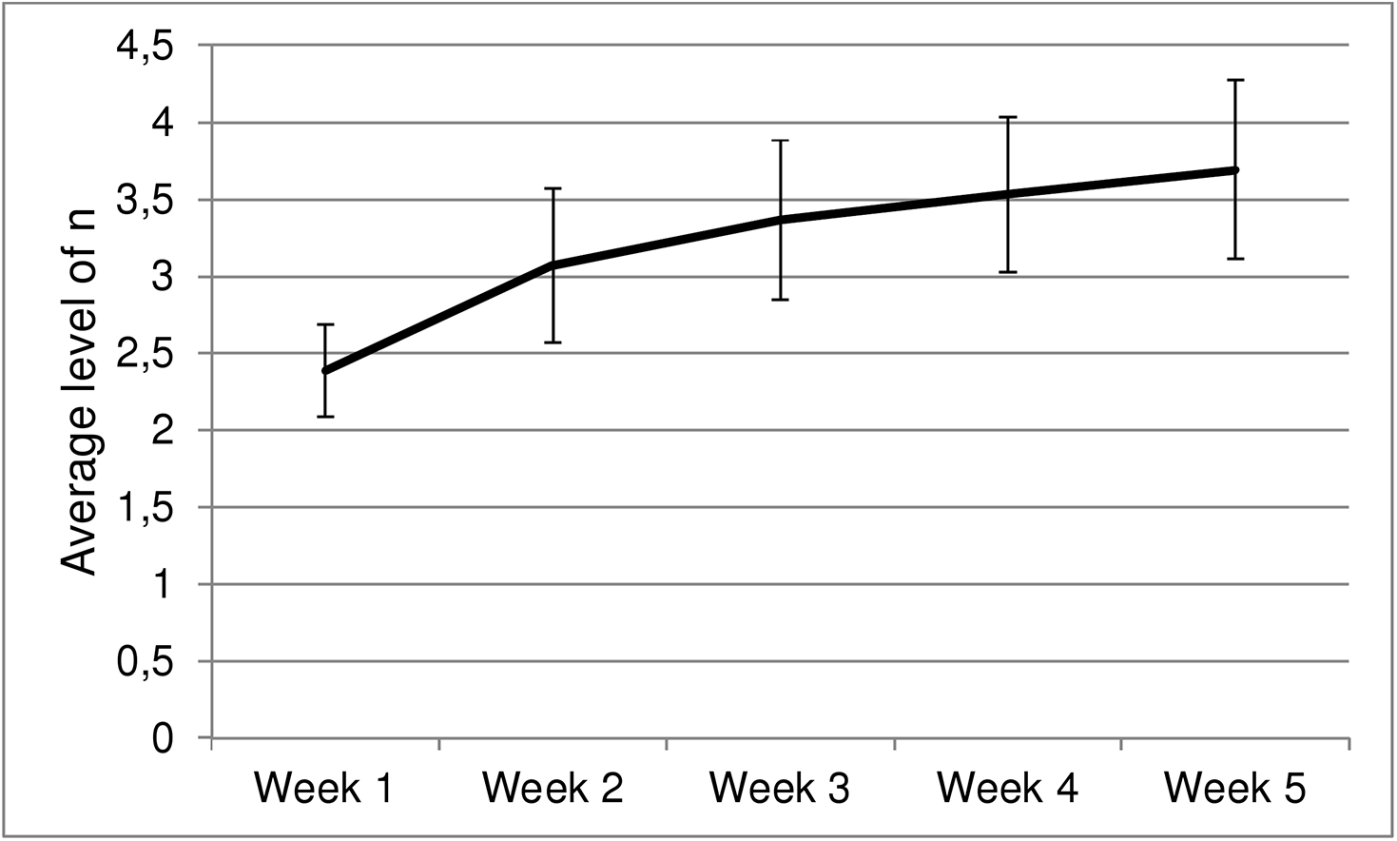
The weekly average level of n achieved in the dual n-back training task. Error bars represent +/- 1 SD.

Nine difficulty levels were created, with the matrix size (3x3 or 4x4), number of turns (4–7) and number of figures (2–5) generally increasing as the levels progressed. The training session details regarding phases, advancement to new difficulty levels, and the reporting of results were identical to that of the SUTT.

Every training session began with the task instructions that were visible until the participant pressed a button on the keyboard. Every sequence started with a prompt “The next sequence is about to begin”, which was shown for 2000ms, after which an empty matrix was displayed for 500ms. Then the figures appeared inside the matrix. The figures disappeared after 7000ms, leaving the matrix empty for 500ms, after which the figures started to “move”. The moves were visible for 2000ms, and they were separated by a blank matrix screen (500ms). At the end of the sequence, the text “Where is the” paired with a figure (in random order) above the matrix indicated which figure was to be located. The participants responded by selecting a square with the computer mouse.

#### Dual n-back task

This training task was based on Jaeggi, Buschkuehl, Jonides, and Perrig’s task design [54]. Eight recorded Finnish syllables, spoken by a native Finnish speaker (*dy*, *ki*, *le*, *nä*, *pö*, *ro*, *su*, *ta*), and a matrix with eight visuospatial locations served as stimuli. Each syllable was presented via headphones, and it was synchronized with the presentation of a white square in one of the matrix locations. The participant had to simultaneously judge whether the syllable and/or the location matched the ones presented n trials back (see Fig 4). The participant was to press the left response button if the visuospatial location matched the location presented n trials back, and the right button if the syllable matched the syllable presented n trials back. The participant was to withhold a response if the location or the syllable did not match the appropriate stimulus presented n trials back.

Each fourteen-minute training session consisted of separate blocks. Every block included 20 + n simultaneous syllable-location trials. The first n trials could not be compared to any previous trials and were thus excluded from the data analyses. The difficulty level (i.e., n) was automatically adjusted after every block on the basis of task performance. N was raised if the participant answered correctly on at least 18 of the 20 trials for both stimulus types. N was decreased if more than five errors were made on either stimulus type. N remained unchanged if the participant made between three to five errors on either, or both, stimulus types. Every training session began with a 2-back block. The minimum difficulty level was 1-back and the maximum was 9-back. Every block began with instructions, a reminder of which response buttons to use, and a text indicating the current level of n. The participant started the block by pressing a keyboard button. Every 500ms trial was followed by a 2500ms blank matrix screen. The instruction screen was once again displayed after completion of the current block. When fourteen minutes had passed, and the currently active block was completed, the task ended and a result screen was displayed. The result screen consisted of the highest level of n achieved during the current training session and the number of blocks completed for each level of n. The participants recorded these results in their personal training logs.

### The training tasks for the active control group

The active control group trained with three computer games: *Angry Birds*, *Bejeweled* 2, and *Peggle*. The games were selected due to their assumed limited demands on WM, general appeal to a wide audience, easy instructions, and the possibility to record progression/scores. Each game was played for fourteen minutes during every training session. The participants recorded their scores (Bejeweled 2 in “Endless” game mode, Peggle in “Adventure” game mode) and progression (Angry Birds and Peggle “Adventure” game mode) after each training session in their personal training logs. The progression in Angry Birds and Peggle followed the standard level progression of the games, with the introduction of new game features as the participants advanced. In these two games, the participants were asked to focus on level progression instead of high scores. Bejeweled 2 did not change during the training sessions.

### Statistical analyses

Individual participants who were extreme outliers (i.e., showing a deviation of at least three times the interquartile range) at pretest were removed from the specific statistical analyses on which they deviated. Outliers as well as data loss due to technical issues are reported separately for each measure in the Results. ANCOVAs with posttest performance as the dependent variable and pretest performance as a covariate were the primary method that was used to compare the training effects in the two groups [55].

## Results

### The WM training tasks

#### SUTT

On average, the participants reached difficulty level 4.53 (*SD* = 2.39) by the end of the 15 training sessions. It took on average 3.25 (*SD* = 1.86) training sessions to advance to the next level.

#### Moving Figures

On average, the participants reached difficulty level 5.47 (*SD* = 2.23) by the end of the 15 training sessions. It took on average 2.09 (*SD* = 0.87) training sessions to advance to the next level.

#### Dual n-back

At the end of the training, the maximum level of n attained was on average 5.60, (*SD =* 0.74). To examine dual n-back training gains, daily n-back means were calculated. The daily n-back means were based on the number of blocks trained at each level of n during each day. A weekly mean was then calculated from the daily means. A paired sample t-test revealed a statistically significant improvement between the first and fifth week means, *t*(14) = 9.30, *p* < .001. The fifth week mean was, on average, 1.31 (*SD* = 0.54) levels higher than the first week mean (Fig 5).

### Motivation and subjective level of improvement in training task performance

A 2x2 mixed model ANOVA with time (start vs. halfway) as a within-subjects factor and group (WM training vs. active control) as a between-subjects factor revealed a significant time x group interaction, *F*(1, 29) = 5.09, *p* = .032, η^2^_partial_ = .149. Interestingly, the WM training group showed a decrease in motivation when comparing the retrospective evaluations of motivation at the start of training to the half-way point, while the participants in the active control group evaluated their motivation to have been roughly equal at both time points (see Table 1). The groups did not differ in their subjective estimations of training improvement, *t* < 1.

### Pre- and posttests

#### The SUT criterion task

The data of two participants in the active control group were lost due to technical issues. The ANCOVA on the updating effect revealed a significant main effect of group *F*(1, 26) = 20.89, *p* < .001, η^2^_partial_ = .445, indicating that the WM training group that had practiced this task was more accurate on the update sequences compared to the active control group at posttest (see Table 2).

**Table 2:** Z-scores, accuracy rates, and RTs (in ms) for the outcome measures (means and SDs).

		WM training group		Active control group	
		PRE	POST	PRE	POST
SUT Updating effect		n = 15		n = 14	
Updating effect	Outcome variable	24.9 (8.6)	9.9 (5.4)	26.5 (10.1)	20.8 (8.1)
Baseline	Accuracy (% correct)	97.5 (4.0)	98.5 (2.7)	97.9 (2.4)	97.4 (2.9)
Updating	Accuracy (% correct)	72.6 (10.2)	88.7 (5.9)	71.3 (8.9)	76.6 (9.0)
N-back		n = 15		n = 13	
N-back effect	Outcome variable	11.3 (6.4)	2.3 (4.0)	11.1 (9.0)	5.9 (5.9)
One-back	Accuracy (% correct)	97.6 (1.5)	98.5 (1.3)	96.6 (1.9)	98.1 (1.7)
Three-back	Accuracy (% correct)	86.3 (6.1)	96.2 (3.7)	85.5 (9.3)	92.2 (6.2)
WM Updating Composite		n = 12		n = 16	
Z-score	Outcome variable	0.14 (0.88)	0.66 (1.25)	-0.10 (1.31)	-0.49 (1.31)
VRM	Accuracy (% correct)	67.4 (11.1)	76.7 (13.7)	72.3 (12.1)	74.2 (10.9)
VisMem Updating	Accuracy (% correct)	88.8 (8.5)	93.8 (8.3)	80.1 (15.1)	77.7 (19.1)
Active WM Capacity Composite		n = 14		n = 16	
Total Score	Outcome variable	18.7 (3.7)	20.8 (3.7)	17.3 (2.3)	17.4 (3.3)
CBT Backward	Sequences correct	9.3 (2.5)	10.6 (2.3)	8.6 (1.8)	9.0 (2.3)
Digit Span Backward	Sequences correct	9.4 (2.6)	10.2 (2.6)	8.7 (1.4)	8.4 (2.5)
Passive WM Capacity Composite		n = 15		n = 16	
Total Score	Outcome variable	21.1 (2.9)	22.4 (3.9)	19.1 (2.0)	19.6 (1.9)
CBT Forward	Sequences correct	10.7 (2.1)	11.5 (2.2)	9.7 (1.9)	9.8 (1.7)
Digit Span Forward	Sequences correct	10.4 (2.4)	10.9 (2.8)	9.4 (1.7)	9.8 (1.5)
Set Shifting Composite		n = 15		n = 15	
Z-score	Outcome variable	0.43 (1.42)	-0.57 (1.08)	-0.43 (1.70)	0.57 (2.10)
VisMem	No-switch RT	1055 (181)	1021 (231)	997 (167)	810 (119)
VisMem	Switch RT	1314 (182)	1147 (179)	1175 (202)	1001 (153)
Number-Letter task	No-switch RT	816 (155)	677 (138)	809 (167)	680 (128)
Number-Letter task	Switch RT	1192 (247)	931 (176)	1144 (228)	1017 (237)
CFIT		n = 15		n = 16	
Total score	Outcome variable	15.7 (2.9)	16.4 (2.8)	14.5 (3.2)	15.9 (3.0)
VisMem Dual task Performance		n = 13		n = 14	
Z-score	Outcome variable	0.42 (2.62)	0.91 (2.90)	-0.39 (2.96)	-0.85 (2.67)
Single sorting rule	Sorting (% correct)	91.2 (9.7)	94.0 (7.8)	87.8 (12.3)	94.0 (6.1)
Single sorting rule	Updating (% correct)	77.4 (11.9)	85.8 (15.2)	75.4 (8.7)	70.8 (16.9)
Mixed sorting rules	Sorting (% correct)	91.7 (3.3)	92.0 (10.4)	92.4 (3.6)	92.9 (4.1)
Mixed sorting rules	Updating (% correct)	78.4 (9.2)	87.5 (9.2)	71.7 (15.9)	72.5 (15.9)

SUT, Selective Updating task; VRM, Verbal running memory task; CBT, Corsi Block Task; CFIT, Culture Fair Intelligence Tests.

Outcome variables refer to the measures that were used in the statistical analyses.

#### N-back task

The data of two participants in the active control group were lost due to technical issues and one participant in the active control group was removed from the statistical analysis for being an extreme outlier on pretest 1-back accuracy. The ANCOVA on the n-back effect showed a significant main effect of group, *F*(1, 25) = 6.44, *p* = .018, η^2^_partial_ = .205, with the WM training group being more accurate after training.

#### WM Updating Composite

The WM updating composite consisted of the summed values of the z-transformations of the number of correct items in the Verbal running memory task and part 1 of the VisMem. Three participants in the WM training group were removed from the statistical analysis for being extreme outliers on pretest accuracy in part 1 of the VisMem. The ANCOVA showed a significant main effect of group, *F*(1, 25) = 5.83, *p* = .023, η^2^_partial_ = .189, with the WM training group being more accurate after training.

#### Active WM Capacity Composite

The active WM capacity composite consisted of the sum of correct items (i.e., correct sequences) in the backward versions of the computerized CBT and Digit span tasks. One participant in the WM training group was removed from the statistical analysis due to an apparent misunderstanding of the task instructions during the Digit span backward posttest. The ANCOVA showed a significant main effect of group, *F*(1, 27) = 5.14, *p* = .032, η^2^_partial_ = .160, with the WM training group having a higher score after training.

#### Passive WM Capacity Composite

The passive WM capacity composite consisted of the sumof correct items (i.e., correct sequences) in the forward versions of the computerized CBT and Digit span tasks. All participants were included in the analysis. The ANCOVA on the passive WM capacity score was non-significant, *F*(1, 28) = 2.59, *p* = .119, η^2^_partial_ = .085.

#### Set shifting composite

As the pretest single task accuracy rates were lower than the mixed task no-switch accuracy rates in both the VisMem and Number-Letter tasks (contrary to what is expected and possibly reflecting an ongoing learning process), the mixing costs were not analyzed. Moreover, as the average error rate in the pretest Number-Letter task was low (1.2% in no-switch and 3.6% in switch trials), accuracy rates were not compared. Hence only the switching cost RT was analyzed. The set shifting composite consisted of the summed values of the z-transformations of the switching cost RTs in the VisMem part 3 and Number-Letter tasks. One participant in the active control group was removed from the statistical analysis for being an extreme outlier on pretest VisMem no-switch sorting accuracy. The ANCOVA on the switching cost RT revealed a significant main effect of group, *F*(1, 27) = 18.46, *p* = .001, η^2^_partial_ = .406, with the WM training group showing a greater decrease in the switching cost RT.

#### Dual task performance

Parts two and three of the VisMem were used as a measure of dual task performance (see the VisMem task). This measure consisted of the sum of the z-transformations of the number of correct items in the running memory measures of the VisMem parts two and three and the total sorting accuracy rates in the same tasks. One participant in the control group was removed from the statistical analysis due to a technical malfunction. Additionally, one participant in the control group and two participants in the WM training group were removed from the statistical analysis for being extreme outliers on pretest accuracy rates. The ANCOVA on dual task performance was non-significant, *F*(1, 24) = 2.03, *p* = .167, η^2^_partial_ = .078.

#### CFIT

No data were excluded from the CFIT analysis. The ANCOVA on the CFIT score was non-significant, *F* < 1.

## Discussion

We set two main aims for the current study: 1) to contribute to the ongoing debate on the transfer effects of WM training and 2) to evaluate whether the magnitude of transfer gain effect sizes of the pre-post measures could be related to the cognitive overlap between the pre-post and training measures. To take into account recent methodological criticism concerning some of the earlier WM training research, we implemented an active control group, an adaptive training regime, and composite scores to measure cognitive constructs. In addition to observing training gains in favor of the WM training group on the criterion task (SUT), near transfer effects were observed on the n-back task, WM updating, and active WM capacity. Additionally, far transfer was observed to set shifting.

A closer inspection of the unexpectedly strong far transfer effect to set shifting revealed that the set shifting composite score was inflated by the VisMem switching cost. The control group improved (i.e., became faster) on both VisMem no-switch and switch trials, while the WM training group "only" became faster on the switch trials (see Table 2). This artificially increased the difference between the groups in the switching cost. The Number-Letter task switching cost did, however, show a small advantage for the WM training group as both groups showed a roughly equal decrease in the no-switch RTs but an unequal decrease in the switch trial RTs. One possible explanation for the VisMem result is that the sorting during this task was performed simultaneously as material was updated in WM (dual-tasking), which possibly confounds the performance on this measure. Another possible explanation is that the improved no-switch response rate is a valid transfer effect in favor of the control group, as fast visual discrimination would seem to be a prerequisite for obtaining a high score in the Bejeweled 2 training game. The visual aspects of the training games might explain the lack of similar transfer effects to the more verbally loaded Number-Letter task. Passive WM capacity, dual task performance, and the CFIT did not show statistically reliable signs of transfer.

**Fig 6:**
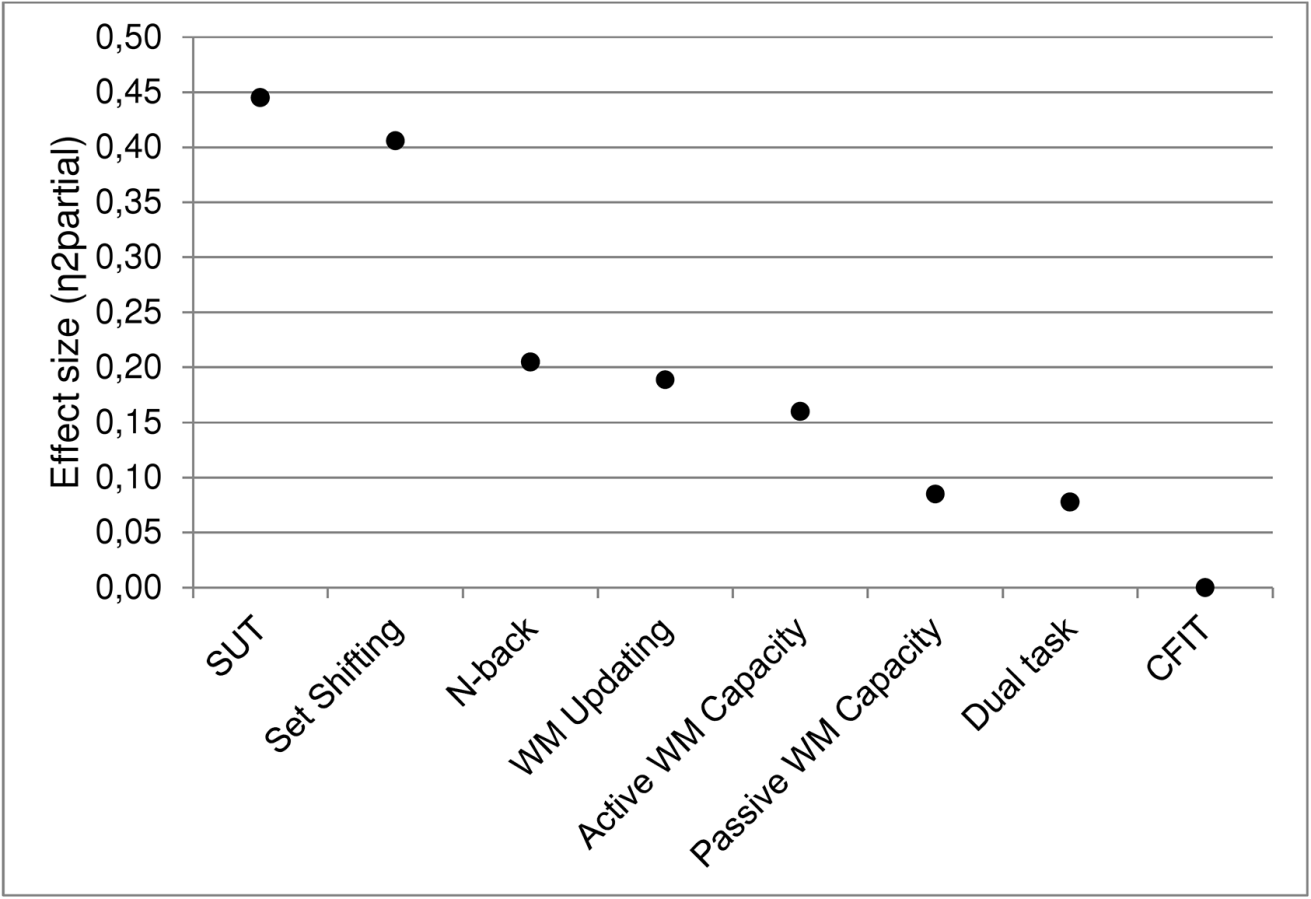
Graphical presentation of the outcome measure effect sizes.

As can be observed in Fig 6, the degree of transfer seems to follow an estimated cognitive overlap between the transfer and WM training tasks. Naturally enough, the strongest gains favoring the WM training group is observed on the trained criterion task (SUT). As discussed above, the largest transfer effect to the set shifting measure is inflated and does not represent the transfer effect accurately. The next one is the n-back, which is not surprising as it is structurally similar to one of the training tasks (the dual n-back task), albeit the stimulus materials were different. The n-back is followed by the WM updating measure, concurring with the fact that the training regime focused on that aspect of WM function. Active and passive WM capacity show the fifth and sixth largest effect sizes, respectively. This also appears logical: albeit the training regime did not focus on capacity per se, it required storage and processing of increasing amounts of information as the training progressed. The dual-tasking far transfer measure showed a smaller effect size than the near transfer measures, which is not surprising considering the limited evidence of far transfer in the WM training literature. Finally, the CFIT measure did not reveal any transfer effect. Even though some of the differences between the effect sizes of the WM transfer measures are quite small, the overall pattern of results support the notion that greater cognitive overlap produces stronger transfer effects. For clinical interventions this would seem to suggest that training regimes that better simulate everyday demands on WM function could be more effective in producing “real world” results.

An important next step would be to study the cognitive overlap between commonly used tests of cognition, as the operationalization of near and far transfer are in many cases primarily based on the face validity of the pre-post tasks (this study included). Careful task analyses on cognitive overlap, for example, by using latent-variable analysis, could provide a principled way to predict generalization following WM training [38]. Some commonly used measures of WM have been shown to correlate weakly at the task level [56], while the correlations are significantly higher at a latent level [38]. The seemingly strong sources of task-specific variance might therefore account for some of the previous inconsistent findings in the training literature. Secondly, studying the effects of systematic manipulations of task features on response patterns can help in teasing apart task components. Thirdly, measuring brain activation during task performance can provide independent evidence on the degree of overlap between a set of tasks [14].

We did not observe transfer to the CFIT following WM updating training, which goes against the meta-analysis results of Au et al. [29]. The lack of transfer to the CFIT might stem from the small sample size in the current study, which makes it difficult to detect small differences between the groups. However, the effect size on the CFIT analysis showed no signs of an emerging group difference (see Fig 6). It could also be related to the specific CFIT subtests that were selected, which might differ somehow from the more commonly used matrix reasoning tasks; however, Au et al. report fairly equal transfer to both matrix and non-matrix reasoning tasks. Lack of transfer to Gf is nevertheless not uncommon, as several other WM training studies have not observed transfer to Gf (e.g., [18, 24, 26, 57]).

Due to practical limitations the group sizes in the current study were quite small, which might have affected the present results. The number of statistical analyses was kept to a minimum in order to minimize the risk of Type I errors. The current study included eight pre-post analyses (or six if the criterion SUT and the problematic set shifting composite are disregarded) of which five (or three) showed training effects in favor of the WM training group. It is also noteworthy that none of the effect sizes were in the opposite direction of what was expected, that is, favoring the active control group. Both training groups retrospectively rated their general motivation to the training to be basically equal. It was in fact the participants in the WM training group that retrospectively reported lower levels of motivation halfway through the training than at the start of training, while the active control group reported no such decrease in motivation. Both groups also experienced an equal subjective improvement in training task performance. Hence it would seem that motivation can be ruled out as a possible confounding factor regarding the observed transfer effects.

In summary, implementing an active control group and an adaptive WM updating training regime, we found that WM updating training resulted in near transfer training effects to a structurally dissimilar n-back task as well as to WM updating and active WM capacity. Importantly, the transfer effects were systematic in that the degree of transfer followed a logical rank order that was based on estimations of relatedness between the transfer and WM training tasks, supporting the notion that greater cognitive overlap produces stronger transfer effects.

## Supporting Information

S1 Dataset (XLSX)
